# Inflammatory Biomarkers of Cardiometabolic Risk in Obese Egyptian Type 2 Diabetics

**DOI:** 10.3390/medsci5040025

**Published:** 2017-11-02

**Authors:** Lamiaa A. A. Barakat, Hassan A. Shora, Ibrahim M. El-Deen, El-Sayed Abd El-Sameeh El-Sayed

**Affiliations:** 1 Biochemistry Department, Faculty of Science, Port Said University, Port Said 065, Egypt; lamiaabarakat@yahoo.com (L.A.A.B.); ieldeen@yahoo.com (I.M.E.-D.); sayedhilab2014@yahoo.com (E.-S.A.E.-S.E.-S.); 2 Internal Medicine Department, Ismailia General Hospital, Ismailia 064, Egypt

**Keywords:** inflammatory biomarkers, obese type 2 diabetics, cardiometabolic risk, insulin resistance

## Abstract

Inflammatory biomarkers provide a minimally invasive means for early detection and specific treatment of metabolic syndrome and related disorders. The objective of this work was to search for inflammatory biomarkers of cardiometabolic risk in obese type 2 diabetics. The study was performed on 165 persons attending the medical outpatient clinic of Ismailia General Hospital. Their mean age was (50.69 ± 10.15) years. They were divided into three groups. The control group was composed of 55 non-obese, non-diabetic healthy volunteers, 32 males and 23 females. Two study groups were included in this study: group 2 was composed of 55 obese, non-diabetic subjects, 25 males and 30 females matched for age and gender. All patients including the control were subjected to clinical history taking, a clinical examination for the measurement of body mass index (BMI). Investigations were carried out for fasting blood glucose, fasting serum insulin, insulin resistance (IR), the lipid profile, lipoprotein band lipoprotein phospholipase A2, and non-high-density lipoprotein cholesterol (non-HDL-C). Urea, albumin and creatinine analysis and liver function tests were performed, and a complete blood count (CBC) was taken. Hemoglobin A1C (HbA1C), serum high-sensitivity C-reactive protein (hs-CRP), interleukin-6 (IL-6) and tumor necrosis factor-α (TNF-α) were tested. There were statistically significant differences among the studied groups in terms of total cholesterol, non-HDL-C, high-density lipoprotein cholesterol (HDL-C), triglycerides (TG), low-density lipoprotein cholesterol (LDL-C), lipoprotein-associated phospholipase A2 and apolipoprotein B. The inflammatory biomarkers hs-CRP, IL-6 and TNF-α were significantly statistically increased in the study groups by (1.62 ± 0.99, 2.32 ± 1.11), (1.73 ± 1.14, 2.53 ± 1.34), and (1.87 ± 1.09, 2.17 ± 0.89) respectively, where *p* < 0.01. Significant positive correlation was found between Homeostatic Model Assessment (HOMA)-IR, hs-CRP and IL-6. There was a significant positive correlation between non-HDL and hs-CRP, IL-6 and TNF-α and triglycerides and hs-CRP. In conclusion, in this study, CRP, IL-6, and TNF-α were significantly elevated in obese Egyptian type 2 diabetics and were positively correlated with insulin resistance, non-HDL and triglycerides. These inflammatory biomarkers could help in the premature identification of obese type 2 diabetic patients at high cardiometabolic risk. Additionally, these biomarkers are critical for providing prognostics and the validity of future potential anti-inflammatory therapeutic modalities.

## 1. Introduction

The World Health Organization (WHO) current estimates of obesity as a global health crisis are that 500 million adults are obese, and 1.5 billion adults are overweight, the majority of which in developing countries [1]. The International Diabetes Federation (IDF) considers type 2 diabetes mellitus (T2DM) to be a major metabolic disease affecting approximately 415 million people worldwide, and the number is expected to reach 642 million by 2040; 90–95% of them have T2DM [2].

Metabolic syndrome increases the risk of cardiovascular disease twofold over a 5–10-year period [3]. It also confers a two- to four-fold increased risk of stroke and a three- to four-fold increased risk of myocardial infarction [4], and it doubles the risk of dying from these catastrophic events in comparison to patients without metabolic syndrome [5,6,7]. T2DM and insulin resistance (IR) are associated with chronic subclinical inflammation and the activation of both the innate and adaptive immune system, but the triggers are unclear [8]. Dysfunctional visceral white adipose tissue due to adipocyte hypertrophy and hyperplasia with faulty remodeling induced by mild hypoxia, increased free fatty acids and metabolic endotoxins leads to increased infiltration by active M1-type tissue macrophages [9]. These M1 macrophages secrete local and systemic proinflammatory cytokines such as interleukin-1B (IL-1B), tumor necrosis factor-α (TNF-α) and interleukin-6 (IL-6) [10]. These cytokines induce insulin resistance in insulin target tissues by activating the suppressors of cytokine signaling proteins; several kinases such as c-Jun N-terminal kinase, IκB kinase β, and protein kinase C; inducible nitric oxide synthase, extracellular signal-regulated kinase, and protein tyrosine phosphatases such as protein tyrosine phosphatase 1B [11]. These inflammatory processes lead to glucolipotoxicity, oxidative stress, and endoplasmic reticulum stress [12]. Ebron et al. [13] reported that a high body mass index (BMI) is associated with increased atherogenic dyslipidemia and insulin resistance in metabolic syndrome; both are linked to low-grade inflammation. These associations are evident by the positive association between BMI, proinflammatory C-reactive protein and IL-6 [13]. High-sensitivity C-reactive protein (hs-CRP) is a circulatory biomarker indicating the existence of inflammation. Multiple studies have shown that hs-CRP is an established risk factor for cardiovascular diseases (CVD) [14,15] and is associated with the progression of T2DM [16]. Yeboah et al. [17] showed that hs-CRP has an additive predictive capability to the traditional Framingham risk score and similar evidence has recommended further measures of hs-CRP in supplemental CVD risk assessment (American College of Cardiology/American Heart Association (ACC/AHA) class IIb-B recommendation), especially among those in the intermediate-risk group (10–20% 10-year CVD risk). Currently, there is no diabetes mellitus (DM) specific risk score that includes hs-CRP as a risk factor. Additional stratification of hs-CRP into low (<1 mg/L), intermediate (1–3 mg/L), and high (>3 mg/L) groups for those with DM has shown incremental predictive values for future CVD events [18].

The role of adipose tissue in metabolic syndrome has continued to evolve with the description of numerous secretory products from adipocytes. These adipokines are important determinants for insulin resistance, either through a traditional (circulating) hormonal effect, or through local effects on the adipocyte. In the mid-1990s, the expression of TNF-α by the adipose tissue of obese rodents and humans was first described. Subsequently, other adipose tissue-derived proteins were described, and many of these adipokines have been implicated in the pathogenesis of the chronic inflammation and insulin resistance associated with obesity [19]. On the basis of this data, inflammatory molecules could be beneficial as biomarkers that provide a minimally invasive means for the early detection and specific treatment of cardiometabolic risk in obese type 2 diabetic patients. Cardiometabolic risk factors account in part, for the ethnic differences in cardiovascular mortality and morbidity. The reasons for racial and ethnic disparities appear to be multifactorial, including genetic inheritance and environment factors (physical inactivity, nutrition, obesity, lower socioeconomic status, smoking, etc. [20]). Furthermore, few studies of cardiometabolic risk biomarkers are conducted in Egypt, so the hypothesis is to evaluate inflammatory biomarkers of cardiometabolic risk in African Arab Egyptian obese type 2 diabetic patients.

## 2. Materials and Methods

This study is a case-controlled study. The samples were collected from the outpatient clinic, Ismailia General Hospital. The biochemical study was done in the laboratory department. The study was approved by the institutional ethical committee of Port Said University (Ethical approval No. 1452, on 19 August 2014). This study was conducted on 165 subjects divided into 3 groups. The control group was composed of 55 non-obese, non-diabetic healthy volunteers, 32 males and 23 females, and their ages ranged from 30 to 79 years with mean age of 51.62 ± 13.42. They were matched for age and sex with the other two study groups. They did not have any clinical condition involving the metabolic or endocrine system. Two other study groups were included in this study. Group 2 was composed of 55 obese, non-diabetic subjects, 25 males and 30 females. Their ages ranged from 30 to 76 years with mean age of 50.69 ± 10.15, and they satisfied the following criteria: high BMI, a negative history suggesting the presence of diabetes, and normal fasting blood glucose. On the other hand, group 3 was composed of 30 obese, diabetic patients (T2DM), diagnosed to be diabetics according to the WHO guidelines. All patients including controls were subjected to clinical history taking and a clinical examination for the following: measurement of BMI (weight in Kg/height in m^2^), waist measurements as measures for central obesity, electrocardiograms and blood pressure, measured while in the supine position on the right arm after a 10-min rest. A standard sphygmomanometer of appropriate cuff size was used, and the first and fifth phases were recorded. The values used in the analysis were the average of three readings taken at 5 min intervals.

### 2.1. Laboratory Investigations

Early morning urine samples were collected. Each urine sample was assayed for creatinine and albumin. The albumin was measured by a latex turbidimetry method. Urine creatinine was measured by Jaffe’s kinetic method. The albumin/creatinine ratio (ACR) was calculated by dividing the albumin concentration (in milligrams) by the creatinine concentration (in mmol)*.* Blood samples were collected, and the following measurements were taken: fasting blood glucose level by the colorimetric method, fasting serum insulin, and insulin resistance by the Homeostatic Model Assessment (HOMA)-IR model (fasting insulin (µU/L) × fasting glucose (nmol/L)/22.5). Also, lipid profile, lipoprotein and lipoprotein phospholipase A2 (Lp-PLA2), non-high-density cholesterol (non-HDL-C) (according to the following: non-HDL-C = total cholesterol − high-density cholesterol (HDL-C)), urea, albumin and creatinine analysis, liver function tests, complete blood count (CBC) by an automated method, hemoglobin A1C (HbA1C), and serum hs-CRP. Blood samples (10 mL of venous blood sample was taken in three tubes, one containing ethylenediaminetetraacetic acid (EDTA), and used for the measurement of the fasting blood glucose level by the colorimetric method), as well as the fasting serum insulin level. The rest of sample was obtained, left to clot and centrifuged to a separate serum that was stored at −20 °C until the time of the assay to avoid loss of bioactive substances. Hemolytic or lipemic samples were completely excluded. Urine samples were taken: random urine collections were obtained from the study subjects and stored in sample cups at 2–8 °C until analysis, before being tested for albumin and creatinine within 36 h.

### 2.2. Statistical Analysis

All the data were analyzed by the Statistical Package for Social Studies (SPSS), version 17 (SPSS Inc., Chicago, IL, USA). The continuous variables were reported as means ± standard deviation (SD), while the categorical variables were presented as percentages. The means were compared using the Student’s *t*-test, and the inflammatory biomarkers of cardiometabolic risk among males and females was determined and compared through a χ^2^-test. As the distribution of biomarkers levels was skewed, the median values of biomarker levels were calculated, and the groups were compared by a median test. Additionally, Spearman’s rank correlation analysis was performed between each biomarker and each cardiometabolic risk, and *p* < 0.05 was considered as statistically significant.

## 3. Results

**Figure 1:**
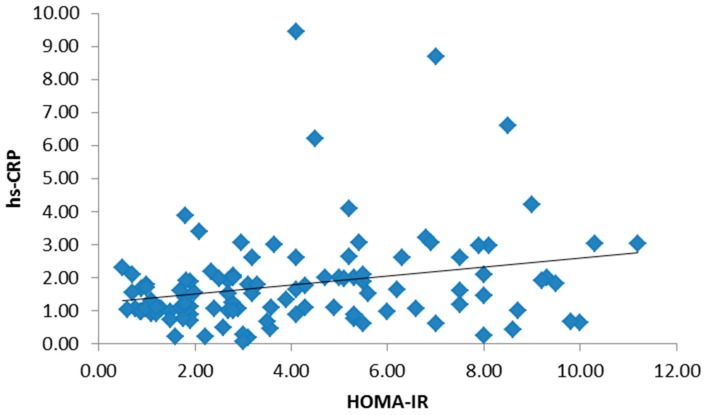
Significant positive correlation between high-sensitivity C-reactive protein (hs-CRP) and Homeostatic Model Assessment-Insulin Resistance (HOMA-IR).

**Figure 2:**
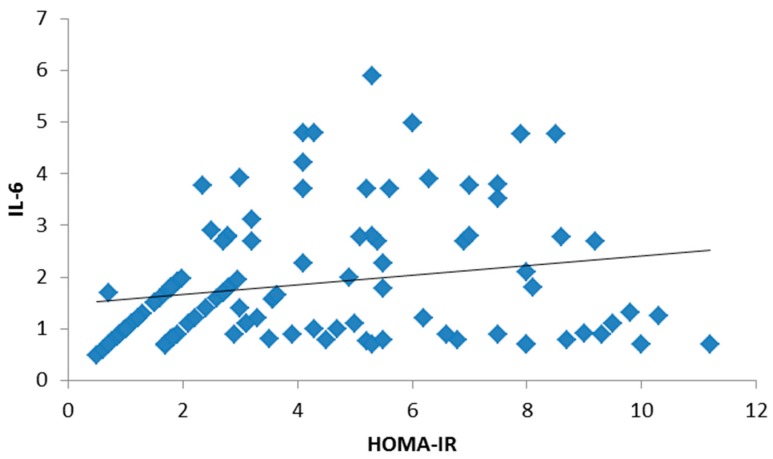
Significant positive correlation between interleukin-6 (IL-6) and HOMA-IR.

**Figure 3:**
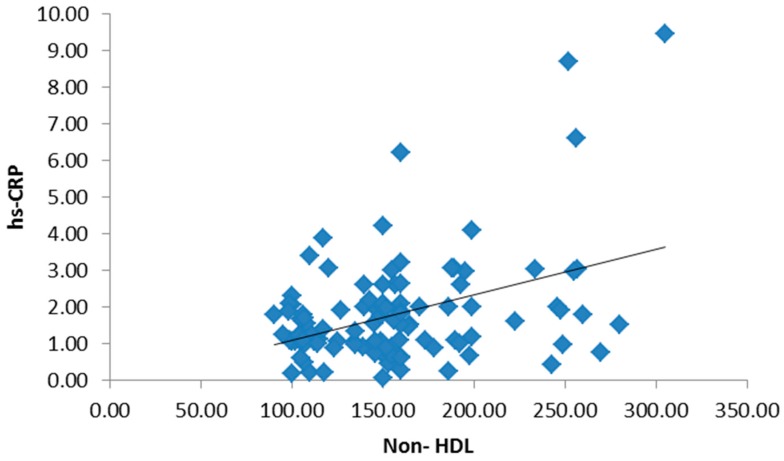
Significant positive correlation between hs-CRP and non-high-density lipoprotein (non-HDL).

**Figure 4:**
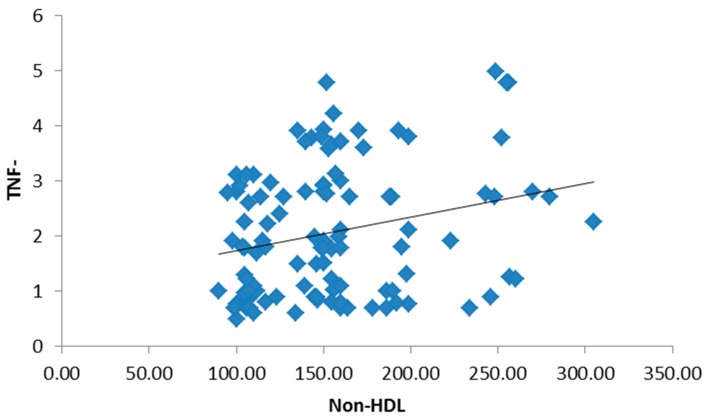
Significant positive correlation between tumor necrosis factor-α (TNF-α) and non-HDL.

There was no significant statistical difference between the three groups regarding age and gender, but there was a statistically significant difference in BMI and waist circumference in both obese type 2 diabetic groups (Table 1). There were no statistically significant differences among the studied groups in terms of hemoglobin (Hb), white blood cells (WBCs), platelets or red blood cells (RBCs). Both the mean systolic blood pressure (122.05 ± 9.14, 132.79 ± 11.63) and diastolic blood pressure (85.12 ± 7.15, 92.92 ± 8.25) were significantly higher in the study groups. Post hoc *p*-values by a Bonferroni test showed a significant difference in systolic and diastolic blood pressure groups I and II (*p* < 0.01), groups I and III and groups II and III (*p* < 0.01). There were statistically significant differences among the studied groups in terms of total cholesterol, non-HDL-C, HDL-C, triglycerides (TG), low-density lipoprotein cholesterol (LDL-C), lipoprotein-associated phospholipase A2 (Lp-PLA2) and apolipoprotein B. Post hoc *p*-values were found by a Bonferroni test. Table 2 showed a significant difference in the parameters of the lipid profile between groups I and II (*p* < 0.01) and groups I and III, and no significant difference between groups II and III (*p* > 0.05). Both liver and kidney function tests are significantly elevated in study groups (Table 3). The inflammatory biomarkers hs-CRP, IL-6 and TNF-α were significantly statistically increased in the study groups (1.62 ± 0.99, 2.32 ± 1.11), (1.73 ± 1.14, 2.53 ± 1.34), and (1.87 ± 1.09, 2.17 ± 0.89), respectively, where *p* < 0.01 (Table 4). There was a significant positive correlation between BMI, hs-CRP and IL-6, and a significant positive correlation between HbA1c and hs-CRP, and IL-6 and TNF-α. Additionally, a significant positive correlation was found between HOMA-IR and hs-CRP as shown in Figure 1. Also, HOMA-IR was significantly correlated with IL-6 (Figure 2) and a significant positive correlation was also found between non-HDL and hs-CRP (Figure 3). Tumor necrosis factor-α (TNF-α) levels were significantly correlated with non-HDL as graphically represented in Figure 4.

## 4. Discussion

Adipose tissue is an organ that was originally thought to simply be a storage organ for triacylglycerol. Recently, it has been recognized as a metabolically active endocrine organ that affects various biological processes, such as energy homeostasis, feeding, immunity, and glucose and lipid metabolism, amongst others. Consequently, various adipokines such as TNF-α, IL-6, leptin, adiponectin, visfatin and others are secreted to the blood stream. Ferretin, hs-CRP, IL-6, TNF-α, and LDL have all been shown to be elevated in those with metabolic syndrome. On the other hand, adiponectin has been shown to be decreased. The association between the different adipokines and CVD risk factors has been under investigation to understand their role in cardiometabolic risk [21].

Thus, a panel of biomarkers that could be used clinically to help predict and establish metabolic syndrome in individuals would be of immense value, not only in treating those that already have the syndrome, but in decreasing the overall prevalence of the disease in the general population. While there have been a number of studies regarding various cytokines and adipokines thought to act as biomarkers for the syndrome, a panel that can be used in clinical practice does not exist. Some have been shown to have a greater potential than others, but no single biomarker has been shown to be indicative of metabolic syndrome [22].

The main objective of this study was to identify metabolic biomarkers that are associated with traditional cardiovascular disease risk factors and MetS in the Egyptian population in the Suez Canal Area. We have studied three groups, including a healthy, non-obese control group (group 1), an obese group (group 2), and an obese diabetic group (group 3). All group subjects selected were matching in age and sex, thus there were no significant differences among the groups in terms of the mean of the age and sex distributions.

In the present study, BMI was statistically significantly higher in each of the obese groups (groups 2 and 3) compared to the control group. The current study has showed that the waist circumference was significantly higher in the obese diabetic group than in the non-obese control group. This was in accordance with Reilly et al. [23], who reported that those with “apple-shaped” bodies (with more weight around the waist) face more health risks, including diabetes. Additionally, the waist circumference was significantly higher in the healthy obese group than in the non-obese control group. This means, according to Reilly et al., that these obese patients may be prone to the development of health problems, including diabetes [23].

In this study, both measures of blood pressure, systolic and diastolic, were assessed in the three studied groups; there was a significant increase seen in both values in obese subjects in comparison with the controls, as well as in obese diabetic subjects in comparison with obese subjects. The factors generally considered responsible for obesity-related blood pressure elevation include an enhanced sympathetic tone, activation of the renin-angiotensin system (RAS), hyperinsulinemia, structural changes in the kidney, and elaboration of adipokines [24].

Our results were similar to findings from other studies, which confirmed the association between obesity and elevated blood pressure [25], and they were also similar to results of Landsberg and Molitch, who stated that diabetes mellitus is closely associated with the prevalence of hypertension [26]. Diabetes and hypertension frequently occur together [27]. There is substantial overlap between diabetes and hypertension in etiology and disease mechanisms [28]. Obesity, inflammation, oxidative stress, and insulin resistance are thought to be the common pathways. Recent advances in the understanding of these pathways have provided new insights and perspectives [29].

Our results showed a significant positive correlation between glycemic control as expressed by HbA1C and liver function markers: alanine aminotransferase (ALT) and aspartate aminotransferase (AST). Similar associations between transaminase levels and diabetes have been shown [30]. Although classic serum markers for liver damage were not elevated above normal ranges, higher values may still indicate metabolic alterations or even injury by diabetes mellitus.

Some renal function parameters were also evaluated in this study, such as serum urea, creatinine, albumin and the urine ACR. Our results showed a significant difference in these parameters in obese diabetic patients in comparison with the non-diabetic obese and non-obese subjects. A 2014 systematic review and meta-analysis of 33 observational studies (including 20 cross-sectional and 13 prospective studies and involving nearly 64,000 individuals) examined the relationship between non-alcoholic fatty liver disease (NAFLD) and the risk of chronic kidney disease (CKD) [31]. NAFLD was diagnosed by biochemistry, imaging or histology; and CKD, by either glomerular filtration rate (GFR) <60 mL/min/1.73 m^2^ or proteinuria. The results of this meta-analysis showed that NAFLD was associated with a nearly twofold increase in the prevalence and incidence of CKD. Similarly, although only a few studies used a liver biopsy to diagnose NAFLD, the presence of histologically confirmed non-alcoholic steatohepatitis (NASH) was associated with an approximately 2.5-fold increased prevalence and incidence of CKD rather than simple steatosis. Moreover, the presence of advanced hepatic fibrosis was associated with a remarkably greater prevalence and incidence of CKD rather than non-advanced fibrosis. In all of these analyses, the significant association between NAFLD and CKD persisted after adjustment for pre-existing diabetes, hypertension and other cardiorenal risk factors [32].

Amartey et al. [33] also showed increased serum urea and creatinine in diabetic subjects [33]. Diabetics are more prone to experiencing kidney dysfunction than non-diabetics. Serum albumin and ACR were evaluated in previous studies by Momin et al. [34], who showed an increase in the ACR and a decrease in serum albumin in diabetics. These results were similar to ours.

Although the 24 h urine collection was previously the gold standard for the detection of microalbuminuria, it has been suggested that screening can be more simply achieved by a timed urine collection or an early morning specimen to minimize changes in urine volume that occur during the day. Use of the ACR in a timed urinary sample is now recommended as the preferred screening strategy for all diabetic patients.

The association of HbA1c with the ACR was assessed. There was a significant correlation between HbA1c and ACR. This is in agreement with other studies [35]. This association might be related to poorly controlled DM leading to renal impairment. There was no significant correlation between HbA1c and serum urea, indicating that the ACR is a better marker for early diabetic nephropathy than serum urea. Thus, raised HbA1c in monitoring DM calls attention to renal function tests to accomplish early diagnosis of preventable renal impairment.

This study showed no significant difference in WBCs, RBCs, platelet count, or Hb among the studied groups, just as no significant difference was found between diabetics and non-diabetics in WBCs or platelet count in the study conducted by Nada [36]. This was in agreement with our results, except for the RBC count and Hb, which were significantly decreased in contrast to this study.

In this study, there was a significant increase in total cholesterol, LDL-C, non-HDL-C and triglycerides in obese subjects with and without diabetes, while HDL-C was significantly decreased. The significant differences in lipid profile markers between the healthy and obese non-diabetic groups was in agreement with the study of Khan and Khaleel [37], who conducted a study on the Saudi population, while the study of Songa et al. [38] showed no significant differences in lipid profile markers between healthy and obese non-diabetic groups. This was not in accord with the current results. However, both studies and many others similar to the study of Yadav et al. [39] agree with these results regarding the significant difference in the lipid profile between diabetic patients and controls.

Lipid profile parameters of a significant difference in obese diabetic and non-diabetic subjects could be explained by the fact that the insensitivity of adipose cells and other target tissues to insulin (insulin resistance), clearly seen in obesity and T2DM, results in dysregulation of enzymes such as lipoprotein lipase, resulting in elevated and extended lipemia due to the failure to rapidly clear plasma triglycerides. In the Veterans Affairs HDL Intervention Trial (VA HIT) study on elderly men, and in the Diabetes epidemiology: collaborative analysis of diagnostic criteria in Europe (DECODE) study that followed more than 10,000 persons, it was found that insulin resistance is positively associated with CVD [40,4,9]. In contrast to these findings, the Strong Heart Study and Framingham Offspring Study have shown a negative association between CVD and insulin resistance [41,42]. Bunerji et al. (2017) proposed a profound understanding of molecular mechanisms of insulin resistance using the biology of energy hemostasis and the precise identification of individuals at risk of CVD for proper drug-targeting [43]. Here, we propose that new revolutionary sciences of proteomics, transcriptomics, metabolomics, pharmacogenomics and bionanotechnology are promising for enforcing a way forward towards achieving breakthrough scientific discoveries and innovative drugs for insulin resistance and to prevent CVD. In diabetic patients, high triglyceride levels and low HDL concentrations are not only proinflammatory [8], but elevated levels of triglycerides rather than hyperglycemia also result in a large release of proinflammatory proteins by adipose tissue contributing to CVD [44]. While dyslipidemia has been studied as a component of the MetS, further studies regarding the chronic inflammatory state derived from this pathologic process are still lacking. Specifically, dyslipidemic abnormalities have mostly been studied in conjunction to T2DM and obesity, but their sole contributory effect has not yet been thoroughly elucidated [45].

It is found that Lp-PLA2 correlated with HbA1c significantly. Our findings were in accordance with the previous studies, in which Lp-PLA2 correlated with HbA1c [46,47] but differed from the results of Jeanenne et al., who found that plasma Lp-PLA2 activity did not appear to be associated with HbA1C [48]. Whether the oxidative stress associated with diabetes is directly attributable, in part, to Lp-PLA2 activity is not known [49]. In a recent study involving an animal model of diabetes and hypercholesterolemia, the expression of Lp-PLA2 by bone-marrow-derived leukocytes was significantly up-regulated in the presence of advanced glycation end products [50]. According to the results of the current study, there was an increase in the inflammatory markers (hs-CRP, IL-6 and TNF-α) in the obese group in comparison to the controls. However, this increase was not significant. There was a statistically significant increase in the same markers when comparing diabetic groups to the non-diabetic group; this was in agreement with previous studies. Rajeev G. et al. [51] and An P. et al. [52] stated that inflammation as measured by serum inflammatory markers has been shown to be increased in people with diabetes. There was a significant correlation between hs-CRP, IL-6 and TNF-α with measures of obesity such as BMI and WC. The clear relationship that we observed between inflammatory marker concentration and BMI shows the role of adipose tissue in initiating and sustaining subminimal inflammation [53]. This agrees with the results of other studies, such as the study of Bastard et al. [53] in which 14 obese women were studied again after 3 weeks of a very low-calorie diet. The diet resulted in a mean reduction of 2.1 kg/m^2^ in BMI and a mean reduction of 3 kg in adipose tissue mass, and it was associated with a significant decrease in IL-6 and TNF-α [54]. However, the clear demarcation between these marker levels in obese diabetics in comparison to obese non-diabetics provides evidence for a positive association between hs-CRP, IL-6 and TNF-α levels and T2DM among our studied population, and also that this association was independent of BMI and WC, suggesting that this elevation might not be limited to obesity alone in this population. Similar observations have been made in previous studies [55,56]. However, in a Prevention of Diabetes and Obesity in South Asians (PODOSA) trial second analysis, weight loss or a decrease in waist circumference via lifestyle intervention showed no impact on biomarkers of cardiometabolic risk [57].

New opportunities are presented when discovering novel inflammatory biomarkers such as a clinical precise marker for determining insulin resistance and residual cardiovascular risk, and monitoring new anti-inflammatory drugs to treat CVD remains a major enigmatic problem [58]. Ridker et al very recently demonstrated the efficacy of anti-inflammatory therapy targeting the interleukin-1β innate immunity pathway with canakinumab, at 150 mg dose every three months, to significantly reduce rate of recurrent cardiovascular events, compared to placebo independent of lowering lipid level, as shown in Reduction in Recurrent Major CV Disease Events (CANTOS) trial [59,60]. Other trials such as the Cardiovascular Inflammation Reduction Trial (CIRT) for evaluating low-dose methotrexate, and the Colchicine Cardiovascular Outcomes Trial (COLCOT) with the chronic use of colchicine to reduce CV events post-myocardial infarction [61,62]. Targeting IL-6 may also be a useful future therapeutic modality to reduce CV events.

## 5. Conclusions

In this study, CRP, IL-6, and TNF-α were significantly elevated in obese Egyptian type 2 diabetics and positively correlated with insulin resistance, non-HDL and triglycerides, key components of metabolic syndrome. Metabolic syndrome is a condition with genetic and acquired etiologies that results in an increase in cardiovascular risks; those with a known and predictable association with metabolic syndrome can provide a means to detect those at risk and allow for intervention as necessary. Inflammatory biomarkers of cardiometabolic risk have a critical role in diagnostic, therapeutic, and prognostic decision-making, particularly in the context of inadequate quantitative risk assessment available for clinicians. This could significantly decrease the burden of complications imposed on patients and the healthcare system via planning to reduce modifiable risk factors. This study is one of the first to investigate the association of inflammatory biomarker levels with cardiovascular risk factors and metabolic syndrome in the Egyptian population.

## Figures and Tables

**Table 1:** Body measurements of the studied groups expressed as means ± standard deviation (SD).

Variable	Group I (*n* = 55)	Group II (*n* = 55)	Group III (*n* = 55)	F	*p*-Value
WC (cm)	80.04 ± 9.68	114.13 ± 14.18	111.1 ± 12.53	30.3	<0.001
BMI (kg/m^2^)	22.35 ± 2.19	35.6 ± 6.12	34.9 ± 7.33	29.1	<0.001

WC: waist circumference; BMI: body mass index.

**Table 2:** The components of the lipid profile among the studied groups.

Variable	Group I (*n* = 55)	Group II (*n* = 55)	Group III (*n* = 55)	F	*p*-Value
Cholesterol (mg/dL)	172.18 ± 21.87	231.78 ± 57.78	224.78 ± 42.96	11.76	<0.01
TG (mg/dL)	122.8 ± 24.27	219.25 ± 89.09	221.25 ± 99.58	4.85	<0.01
HDL-C (mg/dL)	51.75 ± 11.73	33.56 ± 8.16	31.52 ± 7.26	20.18	<0.01
Non-HDL-C (mg/dL)	120.43 ± 12.73	198.23 ± 31.08	193.26 ± 37.66	20.18	<0.01
LDL-C (mg/dL)	87.75 ± 12.73	140.12 ± 35.61	143.72 ± 37.66	20.18	<0.01
Lp-PLA2 (nmol/min/mL)	17.95 ± 6.73	22.72 ± 6.01	24.72 ± 5.06	20.18	<0.01
Apolipoprotein B (g/L)	0.96 ± 0.31	1.11 ± 0.34	1.15 ± 0.29	20.18	<0.01

TG: triglycerides; HDL-C: high-density lipoprotein cholesterol; non-HDL-C: non-high-density lipoprotein cholesterol; LDL-C: low-density lipoprotein cholesterol; Lp-PLA2: lipoprotein-associated phospholipase A2.

**Table 3:** Mean ± SD of liver and kidney functions among studied groups.

Variable	Group I (*n* = 55)	Group II (*n* = 55)	Group III (*n* = 55)	F	*p*-Value
AST (U/L)	37.51 ± 14.27	49.99 ± 17.9	54.71 ± 14	6.38	<0.01
ALT (U/L)	32.58 ± 13.53	47.49 ± 10.16	45.38 ± 11.66	5.32	<0.01
GGT (IU/L)	31.17 ± 12.22	43.17 ± 11.37	41.18 ± 9.47	5.37	<0.01
Creatinine (mg/dL)	0.92 ± 0.29	0.99 ± 0.54	2.28 ± 1.54	10.33	<0.01
Urea (mg/dL)	28.73 ± 4.74	27.17 ± 6.54	34.27 ± 5.91	9.8	<0.01
Albumin (g/dL)	4.21 ± 0.34	4.12 ± 0.58	3.34 ± 0.48	7.4	<0.01
ACR (mg/mmol)	10.95 ± 4.73	11.18 ± 7.07	37.81 ± 24.01	8.11	<0.01

AST: aspartate aminotransferase; ALT: alanine aminotransferase; GGT: gamma glutamyl transferase; ACR: albumin/creatinine ratio.

**Table 4:** Mean of high-sensitivity C-reactive protein (hs-CRP), interleukin-6 (IL-6), tumor necrosis factor-α (TNF-α) among the studied groups.

Variable	Group I (*n* = 55)	Group II (*n* = 55)	Group III (*n* = 55)	F	*p*-Value
hs-CRP (mg/L)	1.45 ± 0.73	1.62 ± 0.99	2.32 ± 1.11	7.23	<0.01
IL-6 (pg/mL)	1.65 ± 1.01	1.73 ± 1.14	2.53 ± 1.34	4.85	<0.01
TNF-α (pg/mL)	1.82 ± 0.81	1.87 ± 1.09	2.17 ± 0.89	20.18	<0.01
